# Clinicians’ use of breast cancer risk assessment tools according to their perceived importance of breast cancer risk factors: an international survey

**DOI:** 10.1007/s12687-018-0362-8

**Published:** 2018-03-05

**Authors:** Anne Brédart, Jean-Luc Kop, Antonis C. Antoniou, Alex P. Cunningham, Antoine De Pauw, Marc Tischkowitz, Hans Ehrencrona, Marjanka K. Schmidt, Sylvie Dolbeault, Kerstin Rhiem, Douglas F. Easton, Peter Devilee, Dominique Stoppa-Lyonnet, Rita Schmutlzer

**Affiliations:** 1 Institut Curie, Supportive Care Department, Psycho-Oncology Unit, 26 rue d’Ulm, 75005 Cedex 05 Paris, France; 2 University Paris Descartes, 71 avenue Edouard Vaillant, 92774 Boulogne-Billancourt, France; 3 Université de Lorraine, 2LPN-CEMA, 23 boulevard Albert 1er-BP, 60446–54001 Cedex Nancy, France; 4 Centre for Cancer Genetic Epidemiology, Department of Public Health and Primary Care, Worts Causeway, CB1 8RN, University of Cambridge, Cambridge, UK; 5 Institut Curie, Cancer genetic clinic, 26 rue d’Ulm, 75005 Paris Cedex 05, France; 6 Department of Medical Genetics, University of Cambridge, Box 238, Level 6 Addenbrooke’s Treatment Centre Cambridge Biomedical Campus, Cambridge, CB2 0QQ UK; 7 Department of Clinical Genetics, Laboratory Medicine, Office for Medical Services and Department of Clinical Genetics, Lund University, 221 85 Lund, Sweden; 8 Netherlands Cancer Institute, Division of Molecular Pathology, Plesmanlaan 121, 1066 CX Amsterdam, The Netherlands; 9 CESP, University Paris-Sud, UVSQ, INSERM, University Paris-Saclay, 16 avenue Paul Vaillant-Couturier, 94807 Villejuif, France; 10 Familial Breast and Ovarian Cancer Centre, Cologne University Hospital and Faculty of Medicine, Kerpener Str. 34, I 50931 Cologne, Germany; 11 Department of Human Genetics, Department of Pathology, Leiden University Medical Centre, S4-P P.O. Box 9600, 2300 RC, Leiden, The Netherlands

**Keywords:** Breast cancer risk assessment, Factors, Tools, BOADICEA, Survey, Clinical practice

## Abstract

The BOADICEA breast cancer (BC) risk assessment model and its associated Web Application v3 (BWA) tool are being extended to incorporate additional genetic and non-genetic BC risk factors. From an online survey through the BOADICEA website and UK, Dutch, French and Swedish national genetic societies, we explored the relationships between the usage frequencies of the BWA and six other common BC risk assessment tools and respondents’ perceived importance of BC risk factors. Respondents (*N* = 443) varied in age, country and clinical seniority but comprised mainly genetics health professionals (82%) and BWA users (93%). Oncology professionals perceived reproductive, hormonal (exogenous) and lifestyle BC risk factors as more important in BC risk assessment compared to genetics professionals (*p* values < 0.05 to 0.0001). BWA was used more frequently by respondents who gave high weight to breast tumour pathology and low weight to personal BC history as BC risk factors. BWA use was positively related to the weight given to hormonal BC risk factors. The importance attributed to lifestyle and BMI BC risk factors was not associated with the use of BWA or any of the other tools. Next version of the BWA encompassing additional BC risk factors will facilitate more comprehensive BC risk assessment in genetics and oncology practice.

**Electronic supplementary material:**

The online version of this article (10.1007/s12687-018-0362-8) contains supplementary material, which is available to authorized users.

## Introduction

Breast cancer (BC) is a major public health problem for women with almost 1.7 million new BC diagnoses and 521, 900 BC deaths estimated worldwide in 2012 (DeSantis et al. 2015).

A number of BC risk factors have been identified, including family history, breast density on mammogram, hormonal exposure, reproductive history and lifestyle. A family history of BC suggests the presence of an inherited genetic variant such as those in the *BRCA1* and *BRCA2* genes which confer a “high” BC susceptibility (Couch et al. 2014). Recently, additional BC genetic risk factors have been identified, including rare variants in genes such as *PALB2*, *CHEK2*, *ATM* (Lee et al. 2016) associated with “moderate” to “high” risk and common “low-risk” variants (Kurian et al. 2016). Non-genetic BC risk factors include hormonal BC risk factors (e.g. use of hormone-replacement therapy, oral contraception), reproductive BC risk factors (e.g. age of first pregnancy, breastfeeding, age at menarche, age at menopause) and lifestyle BC risk factors (e.g. obesity, physical activity, alcohol consumption) (Harvie et al. 2015).

Several models and tools have been developed to enable healthcare professionals to assess the probability of developing BC and/or of carrying a deleterious mutation. These models account for the individual or combined effects of different BC risk factors (Antoniou et al. 2008; Amir et al. 2010; Meads et al. 2012; Quante et al. 2012; Kurian et al. 2016; Cintolo-Gonzalez et al. 2017). They can be used in clinical practice to appraise needs for referral for genetic counselling and testing and to inform decisions on risk management options such as enhanced screening, risk-reducing surgical interventions or chemoprevention (Padamsee et al. 2017). These models differ according to the specific BC risk factors they incorporate, their developmental method and clinical applications.

We report the results of a survey performed as part of the BRIDGES research program (http://cordis.europa.eu/project/rcn/193315_en.html) that aims to inform the development and implementation of a comprehensive genetic test in BC risk assessment and to combine this with additional non-genetic BC risk factors. The latter will be achieved through further development of the Breast and Ovarian Analysis of Disease Incidence and Carrier Estimation Algorithm (BOADICEA) model and BOADICEA Web Application (BWA) (Lee et al. 2014, 2016).

Many BC risk assessment tools are currently available. However, it is unclear which clinicians use them, why they use them and whether these tools fulfil their requirements. In particular, we wished to assess the clinical acceptability of the BOADICEA model and the BWA. In a previous article (Bredart et al. 2018), we reported on clinicians’ perceptions of the BOADICEA model and BWA v3 tool in terms of usability (e.g., data entry timing, risk communication format). In this study, we explored the relationship between the perceived importance attributed to BC risk factors by professional profile, in relation to respondents’ knowledge and usage frequencies of the BWA as well as of six other BC risk assessment tools commonly used in family cancer clinics. The results of our analyses will help to identify perceived deficiencies in the BWA and guide further software development, so that the next version of the tool will enable more comprehensive BC risk assessment in genetics and oncology practice.

## Methods

This was a cross-sectional study based on an international survey.

We conducted an online survey (Bredart et al. 2018) involving one reminder using the LimeSurvey application (http://www.limesurvey.org) (LimeSurvey Project Team, Carsten Schmitz 2015) during May to September 2016.

The survey targeted clinicians who were among the potential 7500 individuals who registered to use the BWA since 2007. In addition, BRIDGES investigators were asked to contact members of their national genetics societies (NGS) to invite them to complete the survey (if they had not already responded through the BOADICEA website). A total of 225, 170, 37 and 32 individuals were contacted from the British, French, Dutch and Swedish NGS, respectively.

### Survey development

The survey questions addressed the following: (1) practice in genetic counselling and testing for cancer predisposition; (2) importance attributed to BC familial, hormonal, reproductive and lifestyle BC risk factors; (3) knowledge/usage frequency of the Gail (Gail et al. 1989), Claus (Claus et al. 1991; Parmigiani et al. 2007), Myriad (Shattuck-Eidens et al. 1997; Frank et al. 2002), BRCAPRO (Parmigiani et al. 1998; Mazzola et al. 2015), IBIS (International Breast Cancer Study) (Tyrer et al. 2004; Quante et al. 2012; Brentnall et al. 2015; Cuzick et al. 2016) and BWA (Antoniou et al. 2004, 2008; Cunningham et al. 2012; MacInnis et al. 2013; Lee et al. 2014, 2016) BC risk assessment tools, and Manchester Scoring System (MSS) (Evans et al. 2004, 2005) and data entry timing using these tools; and (4) socio-demographic and professional background (Supplementary material A).

The questionnaire was developed in line with BRIDGES program objectives, which specified an assessment of the acceptability of the BOADICEA model and the BWA in clinical practice. As the next BWA update is expected to integrate non-genetic and additional genetic BC risk factors, we assessed the degree of importance attributed to familial, hormonal, reproductive and lifestyle risk factors to BC risk assessment (Harvie et al. 2015). For each BC risk factor, five response options from least to most important were provided (see Table 4).

We assessed BWA use with that of six other BC risk assessment tools that could be used in family cancer clinics, implementing the Gail (Gail et al. 1989), Claus (Claus et al. 1991; Parmigiani et al. 2007), Myriad (Shattuck-Eidens et al. 1997; Frank et al. 2002), BRCAPRO (Parmigiani et al. 1998; Mazzola et al. 2015) or IBIS (Tyrer et al. 2004; Quante et al. 2012; Brentnall et al. 2015; Cuzick et al. 2016) models or MSS (Evans et al. 2004, 2005).

These models focus primarily on family/personal cancer history. Their distinctive features are briefly summarised below and described in a table adapted from Kurian et al. (2016) and Cintolo-Gonzalez et al. (2017) (Table 1). The Gail model (or NCI Breast Cancer Risk Assessment Tool: BCRAT) (Gail et al. 1989) and Claus model (Claus et al. 1991; Parmigiani et al. 2007) are used to compute BC risk (only Gail incorporates both reproductive and familial BC risk factors). The MSS (Evans et al. 2004, 2005) and Myriad I or II models (Shattuck-Eidens et al. 1997; Frank et al. 2002) are used to compute the probability of carrying a deleterious *BRCA1* and/or *BRCA2* mutation based on cancer family history. In addition to familial/personal BC risk factors, the MSS also accounts for the presence of a *BRCA1* or *BRCA2* mutation, breast cancer pathology and cancers other than breast or ovarian. The BRCAPRO (Parmigiani et al. 1998; Mazzola et al. 2015), IBIS (Tyrer et al. 2004; Quante et al. 2012; Brentnall et al. 2015; Cuzick et al. 2016) and BOADICEA (Antoniou et al. 2004, 2008; Cunningham et al. 2012; MacInnis et al. 2013; Lee et al. 2014, 2016) models predict *BRCA1* or *BRCA2* mutation carrier probability and BC risk. In addition, BRCAPRO and BOADICEA predict contralateral breast and ovarian cancer risk (Cintolo-Gonzalez et al. 2017). BRCAPRO incorporates family history, *BRCA1* or *BRCA2* mutation status, molecular markers, mastectomy/oophorectomy and ethnicity factors. IBIS incorporates not only family history, *BRCA1* or *BRCA2* mutation status and breast cancer pathology but also reproductive, hormone replacement therapy (HRT) and body mass index (BMI) BC risk factors. The BOADICEA model allows for extensive family history; tumour pathology such as oestrogen and progesterone receptors and HER2, CK5/6 and CK14 status of BC in family member(s) to be taken into account (Lee et al. 2014). BOADICEA also includes the effects of truncating mutations in PALB2, CHEK2 and ATM (Lee et al. 2016).

**Table 1:** Factors included in common breast cancer risk assessment tools

Output of breast cancer risk assessment tool	Predicting breast cancer risk		Predicting genetic carrier status		Predicting breast cancer risk and carrier status		
Factors	Gail	Claus	Manchester	Myriad	BRCAPRO	IBIS	BOADICEA
Family cancer history	Yes/1st d°	Yes/1st and 2nd	Yes/Extensive	Yes/1st and 2nd	Yes/Extensive	Yes/1st and 2nd	Yes/Extensive
Personal breast cancer (age at)	Yes	Yes	Yes	Yes	Yes	Yes	Yes
BRCA1/2 mutation	No	No	Yes	No	Yes	Yes	Yes
Common low-risk alleles	No	No	No	No	No	No	No
Intermediate-high risk mutations (CHEK2, PALB2, ATM, etc.)	No	No	No	No	No	No	Yes
Residual non-BRCA1/2 familial aggregation	No	No	No	No	No	Yes	Yes
BRCA1/2 risk modification by family history	No	No	No	No	No	No	Yes
Breast cancer pathology associations	No	No	Yes	No	Yes	Yes	Yes
Mammography density	No	No	No	No	No	No	No
Hormonal, lifestyle, reproductive risk factors	Yes	No	No	No	No	Yes	No
*Body mass index*	*No*	*No*	*No*	*No*	*No*	*Yes*	*No*
*Breastfeeding*	*No*	*No*	*No*	*No*	*No*	*No*	*No*
*Age at menarche*	*Yes*	*No*	*No*	*No*	*No*	*Yes*	*No*
*Age at 1st birth*	*Yes*	*No*	*No*	*No*	*No*	*Yes*	*No*
*Age at menopause*	*No*	*No*	*No*	*No*	*No*	*Yes*	*No*
*Oral contraception*	*No*	*No*	*No*	*No*	*No*	*No*	*No*
*Hormone replacement therapy*	*No*	*No*	*No*	*No*	*No*	*Yes*	*No*
*Alcohol consumption*	*No*	*No*	*No*	*No*	*No*	*No*	*No*
*Smoking*	*No*	*No*	*No*	*No*	*No*	*No*	*No*
*Physical exercise*	*No*	*No*	*No*	*No*	*No*	*No*	*No*
Other cancers (non-breast/ovarian)	No	No	Yes	No	Yes	No	Yes
Predicting second cancer risks (contralateral, ovarian)	No	No	No	No	Yes	No	Yes

Italicized entries correspond to non-genetic risk factors

Adapted from Kurian et al. (2016); Cintolo-Gonzalez et al. (2017)

For all seven BC risk assessment tools, we assessed knowledge (‘I don’t know the model’), use frequency on a five-point Likert scale ranging from never to always (see Table 3) and convenience (i.e. the time required for data entry).

A preliminary version of the overall survey was designed following survey method recommendations (Edwards et al. 2009; Cottrell et al. 2015). It was pilot-tested with clinical geneticists, genetic counsellors, gynaecologists, psycho-oncologists, a radiotherapist and a methodologist (*n* = 21).

### Data analysis

**Fig. 1:**
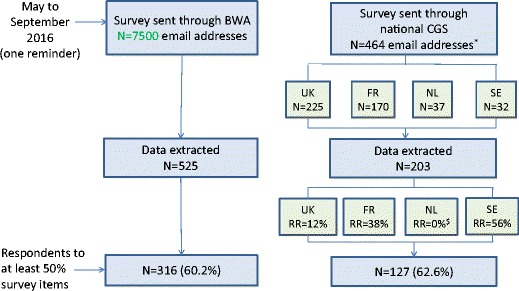
Respondents’ sample

As shown in Fig. 1, 525 and 203 respondents’ data were extracted from the BOADICEA website and NGS survey sources respectively. The response rate obtained from the BOADICEA website survey source could not be estimated as the survey was sent to registered individuals who might no longer use the tool, and tracking the tool use is not legally permitted. Among respondents contacted through NGS, the response rate was 43.7% (203 respondents out of 464 NGS members, excluding those who responded through the BOADICEA website).

Based on psychometric analyses (Husson et al. 2011; Jolliffe 2002), we developed indicators of genetic clinical activity level (i.e. one scale comprising six survey items), and of importance attributed to BC risk factors (i.e. five single items addressing familial and personal cancer history, breast tumour pathology, BMI and breastfeeding), and three multi-item scales corresponding to hormonal BC risk factors (i.e. oral contraception, HRT and exogenous hormone consumption), reproductive BC risk factors (i.e. age at first menstrual period, age at menopause and child bearing at younger age), and lifestyle BC risk factors (i.e. alcohol consumption, smoking and physical activity).

Knowledge and usage frequency were reported in percentages. We computed the number of respondents by the number of BC risk assessment tools used at least occasionally or regularly. Correlation coefficients were calculated between the usage frequencies of the different BC risk assessment tools. We compared the degree of importance attributed to each BC risk factor by health profession (i.e. clinical geneticists, genetic counsellors or nurses and oncology specialists) using a chi-squared test.

Logistic multiple regression analyses (Tabachnick and Fidell 2013) were performed on dichotomized variables addressing knowledge and usage frequency of six of the BC risk assessment tools (excluding Myriad which received less than 100 responses) comparing ignorance of the tool or absent use versus regular or more frequent use.

We explored clinicians’ background characteristics and the degree of importance that they attributed to different BC risk factors as potential explanatory variables, controlling for gender. Age was significantly correlated to respondents’ clinical seniority, so to maintain parsimony, it was not included as an explanatory variable.

In further multivariate analyses, we tested the effect of data entry time for each BC risk assessment tool, including only respondents who use the respective tools. The logarithm of data entry time was computed to allow a normal data distribution.

Statistical analyses were performed with R software version 3.3.1 (R Core Team 2016).

## Results

Overall, 443 respondents completed at least 50% of the survey, comprising 316 (71.3%) and 127 (28.7%) via the BOADICEA website and NGS survey sources respectively (Fig. 1). Due to missing responses, especially in the last sections of the questionnaire, less data on socio-demographics and professional characteristics are available (*N* = 394).

### Sample characteristics

As shown in Table 2, a wide range of geographical regions/countries were represented among respondents, including Southern Europe (28%), Western Europe (21%), UK (20%), North America (18%), Australia (8%) and other countries (6%). Respondents also varied in terms of age and years of clinical experience but were mainly female (77%); most had received a specific training in cancer genetic counselling and testing (70%) and had a clinical activity of 5 to 10 patients per week (42%).

**Table 2:** Sample characteristics (*N* = 394)

Respondents		*N* (%)
Age	20–39	198 (50)
	40–49	82 (21)
	> 50	114 (29)
Gender	Female	305 (78)
Country	Australia	30 (8)
	North America (Canada, USA)	69 (18)
	UK	77 (20)
	Southern Europe (e.g. France, Italy, Spain)	111 (28)
	Western Europe (e.g. Belgium, Germany, Netherlands, Sweden)	81 (21)
	Others (e.g. Argentina, Estonia, India, Israel, Taiwan)	25 (6)
Health profession	Clinical geneticists	115 (29)
	Genetic counsellors/nurses	209 (53)
	Oncology specialists (e.g. gynaecologists, oncologists, surgeons, breast specialists, etc.)	48 (12)
	Others (e.g., genetic lab, (bio) statisticians, etc.)	22 (6)
Seniority/clinical experience	< 6 years	109 (28)
	6–15 years	147 (38)
	> 15 years	130 (34)
Training relevant to genetics	Yes	274 (70)
Level of clinical activity^a^	Low	176 (40)
	Medium	185 (42)
	High	82 (18)

^a^*N* = 443 as this question appeared in the survey beginning and so was answered by more respondents; low = about 5 patients; medium = about 5–10 patients; high > 10 patients/week

## Breast cancer risk assessment tool knowledge and usage frequency

The proportion of participants who did not know one of the BC risk assessment tools included in the survey (excluding the BWA which was reported as not known by two respondents) ranged from 24 (5%) (BRCAPRO) to 61 (14%) (Myriad).

The BWA was used at least occasionally by 413 survey participants (93%) (Table 3). The Gail, Claus, MSS, Myriad, BRCAPRO and IBIS tools were used at least occasionally by 25, 27, 37, 13, 30 and 41% respondents, respectively.

**Table 3:** Breast cancer risk assessment tool knowledge and use frequency among respondents (*N* = 443)

	To estimate breast cancer risk, how often do you use the following gene mutation or cancer risk prediction models? *N* (%)				
Tool (number of users)	Don’t know the model	Never	Occasionally	Regularly	Always
Gail (*N* = 109)	46 (10)	288 (65)	52 (12)	40 (9)	17 (4)
Claus (*N* = 119)	40 (9)	284 (64)	54 (12)	37 (8)	28 (4)
Manchester (*N* = 162)	54 (12)	227 (51)	46 (10)	46 (10)	70 (16)
Myriad (*N* = 56)	61 (14)	326 (74)	36 (8)	12 (3)	8 (2)
BRCAPRO (*N* = 132)	24 (5)	287 (65)	61 (14)	42 (9)	29 (7)
IBIS (*N* = 180)	34 (8)	229 (52)	67 (15)	76 (17)	37 (8)
BOADICEA (*N* = 413)	2 (1)	28 (6)	105 (24)	169 (38)	139 (31)

Regarding the number of tools that were used, the most common observation was the use of one tool at least regularly, by 36% of respondents. The usage frequency of the BWA and other tools was uncorrelated; only the usage frequency of Gail was correlated to the use other tools, i.e. Myriad (*r* = 0.51), Claus (*r* = 0.45), BRCAPRO (*r* = 0.44) and IBIS (*r* = 0.41) (data not shown).

### Breast cancer risk assessment tool data completion time

Over the different BC risk assessment tools, the mean (standard deviation) time in minutes to input clinical data ranged from 3.6 (3.6) (MSS) to 15.6 (10.8) (BWA) (Supplementary material B).

### Perceived importance of breast cancer risk factors

To estimate BC risk, cancer family history was perceived as most important by most respondents (72%). Non-genetic BC risk factors were perceived as most important by between 1.3% (breastfeeding, oral contraception) and 5.4% (age at first menstrual period) of respondents (Table 4).

**Table 4:** Importance of breast cancer risk factors as perceived by clinical geneticists (*N* = 115), genetic counsellors or nurses (*N* = 209) and oncology specialists (*N* = 48)

	For you, how important are the following factors to estimate breast cancer risk?				
	Perceived degree of importance—*N* (%)				
	1 (least)	2	3	4	5 (most)
Familial cancer history**					
Clinical geneticists	1 (0.1)	0 (0.0)	0 (0.0)	22 (19.1)	92 (80.0)
Genetic counsellors/nurses	0 (0.0)	0 (0.0)	5 (2.4)	52 (24.9)	152 (72.7)
Oncology specialists	1 (2.1)	1 (2.1)	1 (2.1)	20 (41.7)	25 (52.1)
Total	2 (0.5)	1 (0.3)	6 (1.6)	94 (25.3)	269 (72.3)
Personal cancer history					
Clinical geneticists	1 (0.1)	3 (2.6)	8 (7.0)	37 (32.2)	66 (57.4)
Genetic counsellors/nurses	0 (0.0)	1 (0.5)	12 (5.7)	83 (39.7)	113 (54.1)
Oncology specialists	1 (2.1)	1 (2.1)	8 (16.7)	18 (37.5)	20 (41.7)
Total	2 (0.5)	5 (1.3)	28 (7.5)	138 (37.1)	199 (53.5)
Breast tumour pathology**					
Clinical geneticists	3 (2.6)	18 (15.7)	32 (27.8)	44 (38.3)	18 (19.8)
Genetic counsellors/nurses	0 (0.0)	10 (4.8)	54 (25.8)	82 (39.2)	63 (30.1)
Oncology specialists	2 (4.2)	4 (8.3)	15 (31.2)	17 (35.4)	10 (20.8)
Total	5 (1.3)	32 (8.6)	101 (27.2)	143 (38.4)	91 (24.5)
Age at first menstrual period					
Clinical geneticists	27 (23.5)	42 (36.5)	34 (29.6)	9 (7.8)	3 (2.6)
Genetic counsellors/nurses	43 (20.6)	62 (29.7)	62 (29.7)	28 (13.4)	14 (6.7)
Oncology specialists	10 (20.8)	13 (27.1)	15 (31.2)	7 (14.6)	3 (6.2)
Total	80 (21.5)	117 (31.5)	111 (29.8)	44 (11.8)	20 (5.4)
Age at menopause					
Clinical geneticists	24 (20.9)	40 (34.8)	37 (32.2)	12 (10.4)	2 (1.7)
Genetic counsellors/nurses	33 (15.8)	59 (28.2)	66 (36.1)	38 (10.2)	13 (6.2)
Oncology specialists	4 (8.3)	14 (29.2)	21 (43.8)	6 (12.5)	3 (6.2)
Total	61 (16.4)	113 (30.4)	124 (33.3)	56 (15.1)	18 (4.8)
Body mass index					
Clinical geneticists	21 (18.3)	42 (36.5)	32 (27.8)	15 (13.0)	5 (4.3)
Genetic counsellors/nurses	34 (16.3)	71 (34.0)	61 (29.2)	35 (16.7)	8 (3.8)
Oncology specialists	2 (4.2)	11 (22.9)	17 (35.4)	13 (27.1)	5 (10.4)
Total	57 (15.3)	124 (33.3)	110 (29.6)	63 (16.9)	18 (4.8)
Child bearing at younger age					
Clinical geneticists	23 (20.0)	40 (34.8)	38 (33.0)	13 (11.3)	1 (0.9)
Genetic counsellors/nurses	29 (13.9)	72 (34.4)	66 (31.6)	30 (14.4)	12 (5.7)
Oncology specialists	6 (12.5)	14 (29.2)	13 (27.1)	11 (22.9)	4 (8.3)
Total	58 (15.6)	126 (33.9)	117 (31.5)	54 (14.5)	17 (4.6)
Breastfeeding*					
Clinical geneticists	21 (18.3)	37 (32.2)	44 (38.3)	12 (10.4)	1 (0.9)
Genetic counsellors/nurses	29 (13.9)	83 (39.7)	63 (30.1)	33 (15.8)	1 (0.5)
Oncology specialists	5 (10.4)	16 (33.3)	14 (29.2)	10 (20.8)	3 (6.2)
Total	55 (14.8)	136 (36.6)	121 (32.5)	55 (14.8)	5 (1.3)
Alcohol consumption****					
Clinical geneticists	34 (29.6)	42 (36.5)	28 (24.3)	7 (6.1)	4 (3.5)
Genetic counsellors/nurses	39 (18.7)	89 (42.6)	56 (26.8)	23 (11.0)	2 (1.0)
Oncology specialists	4 (8.3)	13 (27.1)	14 (29.2)	15 (31.2)	2 (4.2)
Total	77 (20.7)	144 (38.7)	98 (26.3)	45 (12.1)	8 (2.2)
Smoking					
Clinical geneticists	31 (27.0)	38 (33.0)	29 (25.2)	15 (13.0)	2 (1.7)
Genetic counsellors/nurses	42 (20.1)	70 (33.5)	58 (27.8)	31 (14.8)	8 (3.8)
Oncology specialists	15 (31.2)	14 (29.2)	9 (18.8)	6 (12.5)	4 (8.3)
Total	88 (23.7)	122 (32.8)	96 (25.8)	52 (14.0)	14 (3.8)
Oral contraception**					
Clinical geneticists	21 (18.3)	40 (34.8)	42 (36.5)	12 (10.4)	0 (0.0)
Genetic counsellors/nurses	22 (10.5)	57 (27.3)	90 (43.1)	38 (18.2)	2 (1.0)
Oncology specialists	9 (18.8)	18 (37.5)	9 (18.8)	9 (18.8)	3 (6.2)
Total	52 (14.0)	115 (30.9)	141 (37.9)	59 (15.9)	5 (1.3)
Hormone replacement therapy****					
Clinical geneticists	13 (11.3)	27 (23.5)	48 (41.7)	23 (20.0)	4 (3.5)
Genetic counsellors/nurses	6 (2.9)	34 (16.3)	93 (44.5)	66 (31.6)	10 (4.8)
Specialists	1 (2.1)	11 (22.9)	15 (31.2)	11 (22.9)	10 (20.8)
Total	20 (5.4)	72 (19.4)	156 (41.9)	100 (26.9)	10 (2.7)
Physical exercise**					
Clinical geneticists	24 (20.9)	36 (31.3)	41 (35.7)	7 (6.1)	7 (6.1)
Genetic counsellors/nurses	32 (15.3)	76 (36.4)	68 (32.5)	32 (15.3)	1 (0.5)
Oncology specialists	4 (8.3)	14 (29.2)	15 (31.2)	13 (27.1)	2 (4.2)
Total	60 (16.1)	126 (33.9)	124 (33.3)	52 (14.0)	10 (2.7)

Comparison of type of clinicians by the degree of importance attributed to each BC factors: *p ≤ 0.05; **p ≤ 0.01; ***p ≤ 0.001; ****p ≤ 0.0001

Familial history of cancer was perceived as most important significantly more by geneticists than oncology health professionals (*p* ≤ 0.01). More genetic counsellors/nurses than other health professionals considered breast tumour pathology as most important (*p* ≤ 0.01) (Tables 4 and 5).

**Table 5:** Factors associated to breast cancer risk assessment tool use (*N* = 370)—odds ratios [confidence interval]

	Gail	Claus	Manchester	BRCAPRO	IBIS	BOADICEA
Gender (male)	5.15 [2.14–13.6]**	1.26 [0.57–2.80]	0.83 [0.42–1.61]	2.59 [1.26–5.33]**	1.43 [0.68–2.99]	0.80 [0.43–1.48]
Medical profession (Genetic counsellors vs Clinical geneticists)	12.30 [3.17–47.9]***	2.20 [0.97–5.03]	3.22 [1.64–6.27]***	1.22 [0.56–2.63]	8.41 [3.39–20.8]***	0.64 [0.34–1.20]
Medical profession (oncology specialists vs clinical geneticists)	13.60 [2.82–65.2]***	1.06 [0.31–3.65]	0.90 [0.32–2.43]	0.30 [0.07–1.21]	5.47 [1.79–16.6]**	0.48 [0.20–1.15]
Level genetic clinical activity	1.55 [0.94–2.54]	1.26 [0.86–1.84]	0.90 [0.66–1.24]	1.34 [0.93–1.93]	1.36 [0.95–1.97]	0.99 [0.72–1.35]
Experience (6–15 vs < 6 years)	1.13 [0.41–3.10]	1.95 [0.84–4.50]	0.94 [0.52–1.73]	1.48 [0.67–3.26]	1.19 [0.58–2.43]	1.09 [0.58–2.08]
Experience (> − 15 vs < 6 years)	2.97 [1.01–8.79]*	3.06 [1.23–7.54]*	1.48 [0.74–2.95]	1.51 [0.62–3.61]	2.61 [1.12–6.10]*	0.82 [0.41–1.65]
Specific genetic training (Yes)	1.67 [0.60–4.52]	1.23 [0.57–2.71]	1.48 [0.82–2.66]	1.21 [0.56–2.59]	1.77 [0.84–3.72]	0.66 [0.36–1.20]
Family cancer history (more important)	0.59 [0.25–1.40]	1.09 [0.51–2.34]	1.11 [0.62–1.98]	0.71 [0.35–1.46]	1.13 [0.58–2.20]	1.52 [0.87–2.66]
Personal cancer history (more important)	2.66 [1.10–6.46]*	2.56 [1.24–5.26]*	0.80 [0.45–1.43]	1.26 [0.61–2.57]	0.80 [0.41–1.58]	0.49 [0.28–0.86]*
Breast tumour pathology (more important)	0.21 [0.07–0.62]**	0.41 [0.18–0.92]*	1.58 [0.86–2.93]	1.08 [0.50–2.36]	0.63 [0.30–1.35]	3.90 [1.90–7.97]***
Body mass index (more important)	0.10 [0.56–1.75]	0.67 [0.41–1.10]	1.14 [0.77–1.68]	0.87 [0.53–1.40]	1.15 [0.73–1.82]	0.91 [0.61–1.36]
Breastfeeding (more important)	1.28 [0.72–2.28]	1.15 [0.70–1.90]	1.06 [0.72–1.57]	1.02 [0.62–1.66]	0.53 [0.33–0.86]**	0.70 [0.47–1.06]
Reproductive risk factor (more important)^a^	3.06 [1.67–5.65]***	1.88 [1.13–3.11]*	0.57 [0.38–0.86]**	1.63 [0.99–2.69]	4.81 [2.86–8.14]***	0.77 [0.51–1.16]
Hormonal risk factor (more important)^a^	1.18 [0.66–2.14]	0.99 [0.62–1.58]	1.20 [0.84–1.71]	1.16 [0.73–1.87]	0.99 [0.64–1.54]	1.77 [1.20–2.60]**
Lifestyle risk factor (more important)^a^	0.76 [0.42–1.39]	1.17 [0.70–1.94]	1.31 [0.88–1.95]	1.05 [0.63–1.74]	0.63 [0.38–1.02]	1.14 [0.75–1.73]
Nagelkerke pseudo-R2	0.40	0.15	0.15	0.12	0.41	0.14

Never/occasional or do not know = 0 versus at least regular use = 1

^a^Reproductive, hormonal, lifestyle factors include (1) age at first menstrual period, age at menopause, child bearing at younger age; (2) oral contraception, hormone replacement therapy; (3) alcohol consumption, smoking, physical activity, respectively

*p < 0.05; **p < 0.01; ***p < 0.001

Oncology health professionals attributed higher importance to the following BC risk factors than genetics health professionals: breastfeeding (*p* ≤ 0.05), alcohol consumption (*p* ≤ 0.0001), oral contraception (*p* ≤ 0.01), HRT (*p* ≤ 0.0001) and physical exercise (*p* ≤ 0.01).

### Predictors of breast cancer risk assessment tool usage frequency

The professional background characteristics were not associated with the frequency of use of BRCAPRO or the BWA.

Genetic counsellors/nurses and oncology health professionals used the following tools more frequently than clinical geneticists: Gail (OR [CI] = 12.30 [3.17–47.9]; OR [CI] = 13.60 [2.82–65.2]) and IBIS (OR [CI] = 8.41 [3.39–20.8]; OR [CI] = 5.47 [1.79–16.6]). In addition, genetic counsellors/nurses used the MSS more frequently than clinical geneticists (OR [CI] = 3.22, [1.64–6.27]). Longer clinical experience was associated with a more frequent use of the Gail (OR [CI] = 2.97 [1.01–8.79]), Claus (OR [CI] = 3.06 [1.23–7.54]) and IBIS (OR [CI] = 2.61 [1.12–6.10]) tools. The level of clinical activity and specific cancer genetic counselling and testing training was not associated with the use of any of these tools.

To estimate BC risk, respondents who attributed less importance to personal BC (OR [CI] = 0.49 [0.28–0.86]) and more importance to breast tumour pathology (OR [CI] = 3.90 [1.90–7.97]) used the BWA more frequently; the reverse was (unexpectedly) observed for the Gail (personal BC history: OR [CI] = 2.66 [1.10–6.46]; breast tumour pathology: OR [CI] = 0.21 [0.07–0.62]) and Claus (personal BC history: OR [CI] = 2.56 [1.24–5.26]; breast tumour pathology: OR [CI] = 0.41 [0.18–0.92]) tools. The use of the BWA was (unexpectedly) associated with the importance given to hormonal (exogenous) factors (OR [CI] = 1.77 [1.20–2.60]). Considering reproductive BC factors as important was associated with more frequent use of the Gail (OR [CI] = 3.06 [1.67–5.65]), Claus (OR [CI] = 1.88 [1.13–3.11]) and IBIS (OR [CI] = 4.81 [2.86–8.14]) tools and less frequent use of the MSS (OR [CI] = 0.57 [0.38–0.86]). IBIS was used less frequently by individuals who considered breastfeeding as important (OR [CI] = 0.53 [0.33–0.86]).

Among non-genetic BC risk factors, the importance attributed to lifestyle and BMI risk factors to estimate BC risk was not associated with the use of any of these tools.

## Discussion

Respondents to this survey were mostly BWA users (93%), and few were unaware of other BC risk assessment tools commonly used in cancer genetics clinics. Thus, results presented here describe factors that potentially influence the respondents’ choice of using the BWA or another tool.

Uptake of a BC risk assessment tool may depend on its perceived validity, which is related to the type and number of BC risk factors incorporated within the underlying statistical model and any accompanying validation studies. So, in line with the characteristics of the BC risk assessment tools (described in Table 1), respondents who attributed more importance to breast tumour pathology risk factors used the BWA more frequently and used the Gail and Claus tools less frequently. Similarly, those who attributed more importance to reproductive factors used the Gail and IBIS tools more frequently. However, the relationship between some BC risk factors considered important by our respondents and the more frequent use of some tools was unexpected, given that some tools do not include the BC risk factors considered important by their users (perhaps suggesting lack of understanding of the underlying risk model). For example, we found that the use of the BWA was related to the importance attributed to exogenous hormonal BC factors even though this model does not take these BC risk factors into account. In this study, most respondents regularly used only one tool. It is possible that one tool is selected to optimally respond to one’s major clinical needs. For example, the BWA may be used, in spite of not currently accounting for hormonal factors, because it considers a number of familial and genetic factors.

Uptake of a BC risk assessment tool may also depend on the health professional’s patient population and role (i.e. to guide referral to family history clinics, to assess BC risk or the probability of harbouring a deleterious mutation, to make oncology treatment decisions, to advise on cancer risk management). Respondents’ professional background appeared related to the importance attributed to familial or hormonal, reproductive and lifestyle factors. This may explain the distinct uptake of some BC risk assessment tools according to the type of respondents’ profession. Indeed, oncology specialists attributed more importance to hormonal and lifestyle factors and also used the Gail and IBIS tools more frequently than clinical geneticists. However, genetic counsellors and nurses also used these tools more frequently as well as the MSS, which may be related to role sharing between medical and non-medical genetics clinicians. BWA use was not related to the type of health profession or the use of other tools, suggesting that this tool may respond to various clinical needs.

In contrast, the Gail tool was moderately associated with the use of other tools such as Claus, Myriad, BRCAPRO and IBIS. In fact, our respondents were mostly genetics clinicians, and validation studies suggest that Gail is less appropriate to assess BC risk in hereditary high-risk populations [Evans and Howell 2007]. Moreover, Gail does not predict mutation carrier status, so this tool seems to require a complementary assessment tool for professionals in this survey.

Uptake of BC risk assessment tools may be related to usability and ease of data entry (e.g. depending on the amount of information) and accessibility (i.e. availability on the Web and ease with which statistical outputs can be understood and communicated to patients). In this study, only the uptake of Claus and the MSS was related to the usability of the tool (data presented in supplementary material C). In cancer genetics clinics, these tools may be used systematically for all patients to appraise the need for genetic testing and so they must be easy to use. In contrast, it is possible that tools with lower usability (that require more time to enter data) such as the BWA may be acceptable in specific situations, e.g. to refine a BC risk assessment or estimate the risk of contralateral BC and other cancers.

The importance given to lifestyle and BMI BC risk factors was not associated with the use of any tool. This was expected as the BC risks associated with these risk factors are not as high as those associated with genetic risks (Amir et al. 2010; Kurian et al. 2016). Moreover, IBIS was the only tool that takes BMI into account in risk calculations. The presence of a substantial family history of BC and the likelihood of carrying a deleterious mutation are important when predicting the incidence of BC, and thus are the focus of genetics clinicians. However, BMI (Quante et al. 2015; Lecarpentier et al. 2015) and reproductive and hormonal BC risk factors (Lecarpentier et al. 2015) seem to modify the BC risk associated with high-risk genetic variants. Moreover, the influence of lifestyle on BC risk has also been suggested among women at increased risk or with a *BRCA*1/2 gene mutation (Easton et al. 2015). Lifestyle factors may be targeted for change by health interventions to reduce BC risk (Meads et al. 2012). Studies have shown that discussion of these factors with counselees is variable across health professional specialties (Julian-Reynier et al. 2015) and may be infrequent in clinical genetics practice (Albada et al. 2014). As the next version of the BWA will incorporate these BC risk factors, this updated tool may facilitate more comprehensive BC risk counselling.

Our study has several limitations:

1. This survey was conducted via the BOADICEA website and European NGS, and so the respondent sample mainly comprised BWA users and European genetics clinicians. Thus, our findings may not be generalised to other, more diverse clinician populations in other geographic areas. In Canada, for example, general practitioners, specialists and genetic counsellors most frequently used the BRCAPRO and Gail tools, whereas the BWA was less frequently used (by 13% compared to 25 and 18% for the BRCAPRO and Gail, respectively) (Amara et al. 2015).
2. Some BC risk assessment tools investigated were used by a limited number of respondents, and so we may have lacked power to provide reliable estimates of the BC risk factors related to their use. In addition, the overall high usage frequency of BWA in this sample and thus the lack of variation in this tool usage frequency may have also hampered the detection of some relationships. For example, although some experience is needed for proper use of the BWA, more frequent use of some of the investigated tools (but not BWA) was related to increased clinicians’ seniority.
3. The survey design allows us to comment on the associations, but we cannot ascertain the directionality of the relationships, nor their specificity over time, along stages of the BC genetic counselling process.

## Conclusion

Our study provides novel insights into the acceptability of the BWA by analysis of BC risk factors incorporated in this tool and other common BC risk assessment tools. These results suggest that while the BWA does fulfil various clinicians’ needs, it does not incorporate some BC risk factors considered important by its users. The results of our analyses will help to identify perceived deficiencies in the BWA and guide further software development (including integration of additional genetic and non-genetic BC risk factors), so that the next version of the BWA will facilitate more comprehensive BC risk assessment in genetics and oncology practice.

## Electronic supplementary material

ESM 1 (DOC 109 kb)
