# Supplementary material for: Clinicians’ use of breast cancer risk assessment tools according to their perceived importance of breast cancer risk factors: an international survey
Source: J Community Genet. 2018 Mar 5;10(1):61–71. doi: 10.1007/s12687-018-0362-8 (PMC6325038; doi:10.1007/s12687-018-0362-8)
Supplement: Supplementary file 1 — (DOC 109 kb) [file 12687_2018_362_MOESM1_ESM.doc]

# Supplementary material A. Survey content

| **Survey section and examples of items** | **Scoring and psychometric information** |
| --- | --- |
| **Section 1. Practice of genetic counselling and testing for cancer predisposition** |  |
| Counselling for breast or ovarian cancer genetic predisposition | 5 response options: none to more than 20 patients per week.  An overall indicator of clinical genetic activity level was created based on 6 items after multiple correspondence analyses (MCA) [1]. |
| Counselling for other cancer genetic predisposition |
| Ordering a genetic test in case of breast or ovarian cancer predisposition |
| Disclosing cancer genetic test results |
| Ordering a genetic test for breast cancer treatment decision making |
| Ordering a genetic test for ovarian cancer treatment decision making |
| **Section 2. Breast cancer risk factors perceived importance** |  |
| ***Familial, personal factors*** |  |
| Familial cancer history | 5 response options: Least to most important |
| Personal cancer history |
| Breast tumour pathology |
| ***Hormonal, reproductive, lifestyle factors*** |  |
| 1. Age at first menstrual period | 5 response options: Least to most important  Three factors were evidenced after principal component analysis (PCA) [2] allowing for computing continuous variables related to the importance given to reproductive (items 1, 2, 4); lifestyle (items 5, 6, 9) and hormonal factors (items 7, 8). Item 3 (BMI) was considered a single item. |
| 2. Age at menopause |
| 3. Body mass index (BMI) |
| 4. Child bearing at younger age |
| 5. Alcohol consumption |
| 6. Smoking |
| 7. Oral contraception |
| 8. Hormone replacement therapy |
| 9. Physical exercise |
| **Section 3. Breast cancer risk assessment tools: Reported knowledge, use frequency and data entry timing** |  |
| Gail/NCI model – Breast Cancer Risk Assessment Tool (BCRAT) | 5 response options: Don’t know the model, Never, Occasionally, Regularly, Always.  Time in minutes. |
| Claus/Yale University model |
| Manchester Scoring System |
| Myriad model |
| BRCAPRO model |
| Tyrer-Cuzick model – International Breast Cancer Study (IBIS) |
| Breast and Ovarian Analysis of Disease Incidence and Cancer Estimation Algorithm model (BOADICEA) |
| **Section 4. Socio-demographic and professional background** |  |
| Age, gender, country of clinical practice | Country was categorized based on the number of respondents by country to have at least 6% respondents by category. |
| Declared medical profession | 9 options and other. Due to professional category size, only clinical geneticists, genetic counsellors and specialists (gynaecologist/obstetrician, radiologist, oncology surgeon, breast specialists…) were compared. |
| Clinical seniority | 5-point: 1-5 years to 21 years of more experience in providing patient care, Does not apply |
| Declared specific genetic training | Yes/No |

**Supplementary material B. Data entry mean time (standard deviation) by breast cancer prediction tool use frequency and** by clinical geneticists (N = 115), genetic counsellors or nurses (N=209) versus specialists (N=48)

|  | **Total** | **Occasionally** | **Regularly/Always** |
| --- | --- | --- | --- |
|  | **Mean (SD) data entry timing** | | |
| **Gail (N=104)** * | 4.9 (4.0) | 5.0 (3.3) | 4.7 (4.6) |
| Clinical geneticists | 4.1 (2.6) | 4.1 (2.2) | 4.0 (4.1) |
| Genetic counsellors/nurses | 4.7 (3.7) | 5.5 (3.3) | 4.2 (3.9) |
| Specialists | 6.6 (5.0) | 4.7 (3.4) | 8.3 (5.7) |
| **Claus (N=114)** ^+,§§^ | 7.1 (7.1) | 8.5 (7.3) | 6.0 (6.8) |
| Clinical geneticists | 7.6 (8.2) | 9.0 (10.2) | 5.7 (4.5) |
| Genetic counsellors/nurses | 6.1 (5.7) | 7.4 (5.2) | 5.1 (5.9) |
| Specialists | 15.3 (8.8) | 12.6 (4.9) | 18.0 (11.5) |
| **Manchester (N=157)** ^+,§§^ | 3.6 (3.6) | 4.6 (2.9) | 3.2 (3.8) |
| Clinical geneticists | 2.9 (2.4) | 3.5 (2.7) | 2.6 (2.3) |
| Genetic counsellors/nurses | 2.9 (2.6) | 3.9 (2.3) | 2.7 (2.6) |
| Specialists | 6.7 (5.6) | 5.6 (2.6) | 7.4 (6.9) |
| **BRCAPRO (N=125)** | 9.2 (6.0) | 9.4 (6.2) | 9.0 (5.9) |
| Clinical geneticists | 9.1 (6.3) | 8.3 (3.7) | 9.5 (7.5) |
| Genetic counsellors/nurses | 8.6 (6.1) | 9.4 (7.0) | 7.9 (5.0) |
| Specialists | 11.3 (6.4) | 10.1 (6.5) | 14.3 (6.0) |
| **IBIS (N=173)** ^+^,** | 7.1 (5.1) | 8.7 (6.1) | 6.2 (4.2) |
| Clinical geneticists | 6.9 (4.1) | 7.0 (4.4) | 6.9 (3.5) |
| Genetic counsellors/nurses | 6.8 (5.1) | 9.7 (6.6) | 5.7 (4.0) |
| Specialists | 8.8 (6.2) | 7.6 (8.2) | 9.5 (5.0) |
| **BOADICEA (N=407)** | 15.6 (10.8) | 16.9 (14.4) | 15.2 (9.3) |
| Clinical geneticists | 16.4 (11.9) | 18.3 (13.7) | 15.8 (11.3) |
| Genetic counsellors/nurses | 15.1 (8.7) | 14.7 (7.6) | 15.2 (9.1) |
| Specialists | 15.5 (9.0) | 16.1 (11.7) | 15.2 (7.7) |

+, ++, +++, +++; §, §§, §§§, §§§§; *, **, ***, **** = *p* value ≤ 0.05; 0.01; 0.001; 0.0001 for timing (log of time) by use frequency, type of clinician and interaction between use frequency and type of clinician (clinical geneticist, genetic counsellor/nurse or specialist).

**Supplementary material C. Factors associated to breast cancer risk assessment tools’ use frequency in respondents knowing the tool – β coefficient (Odds Ratios)**

|  | **Gail**  **N=87** | **Claus**  **N=101** | **Manchester**  **N=139** | **BRCAPRO**  **N=103** | **IBIS**  **N=147** | **BOADICEA**  **N=353** |
| --- | --- | --- | --- | --- | --- | --- |
| Gender (male) | 3.19 [0.64-16.13] | 0.82 [0.24-2.75] | 0.44 [0.13-1.50] | 1.79 [0.59-5.44] | 1.93 [0.63-5.97] | 0.80 [0.41-1.55] |
| Medical profession (Genetic counsellors vs Clinical geneticists) | 17.46 [1.79-170.2]* | 3.22 [0.90-11.44] | 1.92 [0.47-7.82] | 0.54 [0.17-1.72] | 10.18 [2.80-36.71]*** | 0.65 [0.33-1.28] |
| Medical profession (Specialists vs Clinical geneticists) | 8.33 [0.98-71.3] | 3.60 [0.58-22.6] | 2.94 [0.39-22.43] | 0.15 [0.03-0.87]* | 11.47 [2.39-55.11]** | 0.50 [0.20-1.26] |
| Level genetic clinical activity | 1.38 [0.62-3.09] | 0.99 [0.55-1.79] | 0.81 [0.40-1.64] | 1.26 [0.69-2.31] | 1.52 [0.91-2.55] | 0.98 [0.71-1.36] |
| Experience (6-15 vs < 6 years) | 1.93 [0.31-12.1] | 3.39 [0.84-13.9] | 0.20 [0.05-0.87]* | 0.87 [0.28-2.71] | 1.43 [0.49-4.20] | 1.13 [0.57-2.24] |
| Experience (>-15 vs < 6 years) | 9.68 [1.11-84.5]* | 3.46 [0.77-15.6] | 0.29 [0.05-1.58] | 1.02 [0.29-3.52] | 1.60 [0.50-5.17] | 0.80 [0.38-1.68] |
| Specific genetic training (Yes) | 0.84 [0.17-4.10] | 0.18 [0.04-0.76]* | 1.42 [0.42-4.72] | 0.69 [0.22-2.18] | 1.04 [0.38-2.89] | 0.64 [0.34-1.23] |
| Family cancer history (more important) | 0.98 [0.25-3.81] | 1.75 [0.58-5.35] | 5.75 [1.85-18.04]** | 1.43 [0.53-3.85] | 0.79 [0.31-2.05] | 1.67 [0.92-3.02] |
| Personal cancer history (more important) | 3.53 [0.78-15.9] | 3.63 [1.25-10.7]* | 0.79 [0.24-2.57] | 1.00 [0.36-2.83] | 0.91 [0.36-2.33] | 0.45 [0.25-0.82]** |
| Breast tumour pathology (more important) | 0.11 [0.02-0.74]* | 0.59 [0.15-2.41] | 1.30 [0.36-4.63] | 1.27 [0.41-4.00] | 0.95 [0.32-2.80] | 4.35 [1.99-9.46]*** |
| Reproductive risk factor (more important) | 3.60 [0.94-13.9] | 1.62 [0.62-4.27] | 0.53 [0.22-1.26]  653.5 | 1.62 [0.66-3.10] | 3.06 [1.42-6.66]** | 0.73 [0.47-1.13] |
| Lifestyle risk factor (more important) | 0.85 [0.33-2.20] | 1.58 [0.75-3.37] | 3.16 [1.21-8.20]* | 1.22 [0.60-2.46] | 0.76 [0.37-1.54] | 1.26 [0.81-1.95] |
| Hormonal risk factor (more important) | 1.97 [0.67-5.74] | 0.89 [0.41-1.95] | 1.16 [0.55-2.46] | 2.05 [1.00-4.20]* | 0.87 [0.44-1.71] | 1.72 [1.14-2.57]** |
| BMI (more important) | 0.98 [0.40-2.44] | 0.84 [0.37-1.90] | 0.92 [0.38-2.24] | 0.61 [0.28-1.32] | 1.01 [0.51-2.02] | 0.94 [0.62-1.44] |
| Breast feeding (more important) | 1.11 [0.42-2.93] | 0.72 [0.32-1.62] | 0.61 [0.25-1.49] | 0.63 [0.29-1.36] | 0.72 [0.37-1.40] | 0.70 [0.45-1.08] |
| Data entry mean time (logarithm) | 0.79 [0.32-1.99] | 0.44 [0.25-0.80]** | 0.39 [0.19-0.82]* | 0.92 [0.49-1.74] | 0.75 [0.39-1.42] | 0.88 [0.58-1.34] |
| **Nagelkerke pseudo-R2=** | **0.53** | **0.28** | **0.38** | **0.17** | **0.33** | **0.15** |

Occasional =0 versus at least regular use=1; ** p < 0.05; ** p < 0.01; *** p < 0.001*. BMI=Body Mass Index.

1. Husson, F., Lê, S., & Pagès, J. (2011). Exploratory multivariate analysis by example using R. Boca Raton: CRC Press
2. Jolliffe IT. Principal Component Analysis, Second Edition, Springer, 2002
